# Case Report: A case of sudden death due to occupational nicotine poisoning: clinical presentation and MRI findings

**DOI:** 10.3389/fphar.2025.1567380

**Published:** 2025-08-25

**Authors:** Jianjian Liu, Zhaozhao Shan, Xiangdong Jian, Baotian Kan

**Affiliations:** ^1^ Department of Poisoning and Occupational Diseases, Emergency Medicine, Qilu Hospital of Shandong University, Cheeloo College of Medicine, Shandong University, Jinan, Shandong, China; ^2^ Department of Occupational and Environmental Health, School of Public Health, Cheeloo College of Medicine, Shandong University, Jinan, Shandong, China; ^3^ Department of Nursing, Department of Gerontology, Qilu Hospital, Cheeloo College of Medicine, Shandong University, Jinan, Shandong, China

**Keywords:** nicotine poisoning, occupational exposure, event, sudden death, post cardiopulmonary resuscitation

## Abstract

**Background:**

Nicotine is a toxic alkaloid commonly found in tobacco products. This paper presents the clinical case of a patient who was exposed to a nicotine-laden waste liquid.

**Case presentation:**

A 24-year-old male arrived at a local hospital in a state of coma and cardiac arrest. Following successful cardiopulmonary resuscitation, he was transferred to our facility for further treatment. Magnetic resonance imaging of the patient’s head and neck revealed bilateral ischemic infarctions in the hippocampal region. After 24 days of treatment, the patient was discharged with significant improvement.

**Discussion:**

This case highlights the potential for severe clinical manifestations to arise shortly after a single exposure to a substantial amount of nicotine.

**Conclusion:**

In clinical practice, it is crucial to promptly assess the patient’s occupational history to identify the cause of the condition, closely monitor vital signs, and provide active supportive and symptomatic care. Additionally, this case underscores the importance of ensuring production safety and increasing awareness regarding occupational exposure risks.

## 1 Introduction

Nicotine is a toxic organic compound found in nightshade plants (Solanaceae) and is known to act as a botanical insecticide (Becam et al., 2023). It takes only 10–20 s for nicotine to enter the body and travel through the bloodstream to the brain, where it binds to nicotinic acetylcholine receptors (nAChRs), triggering a toxic response (Liu et al., 2024). The clinical manifestations of nicotine intoxication depend on the dose: small amounts typically produce excitatory symptoms related to nAChR activation, while larger doses can lead to opposing effects (Scarpino et al., 2020). This paper reports a case of coma and cardiac arrest resulting from significant nicotine exposure in the workplace, where a 24-year-old male arrived at a local hospital in a state of coma, cardiac arrest, and respiratory distress. Physical examination revealed a comatose state, abnormal pupil response, and elevated heart rate. Laboratory results showed acidosis, high white blood cell count, and elevated inflammatory markers. We have discussed several key aspects: the patient’s clinical presentation, abnormal examination findings, treatment approaches, and recovery. Additionally, we also explored the clinical features of nicotine poisoning and the distinctive cranial MRI findings associated with this case. Finally, we emphasized the critical need for caution regarding occupational exposure during industrial production processes.

## 2 Case description

### 2.1 Occupational setting

**FIGURE 1:**
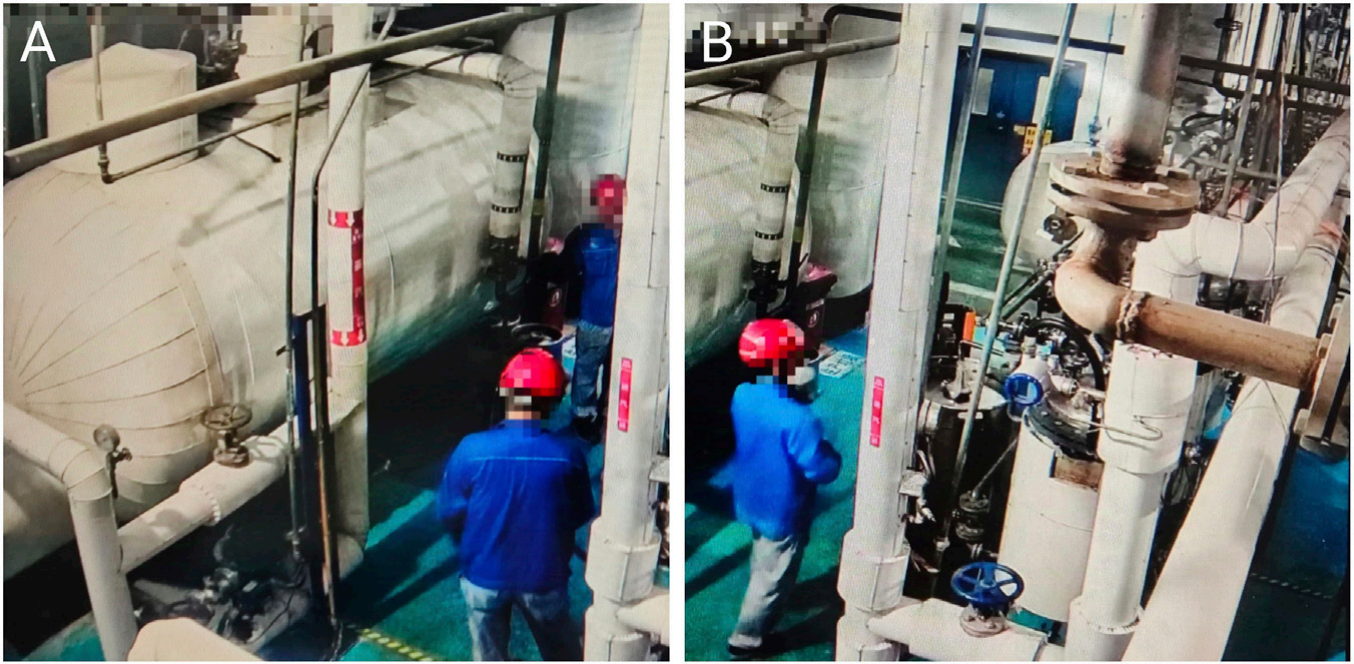
Inside view of the patient workshop **(A,B)**.

The patient is a 24-year-old male employed at a liquid waste treatment plant for a pharmaceutical company. He had been in this position for 2.5 months and was previously in good health. From 24 June 2024, to 12 September 2024, he worked in a workshop responsible for handling waste liquids. His duties included transferring waste liquids into recovery drums. The workshop measures 15 m wide, 20 m long, and 24 m high, featuring a frame structure (see Figure 1). It was equipped with natural ventilation and did not emit any irritating odors. Workers were required to wear rubber gloves and long-sleeved overalls during operations.

### 2.2 Exposure incident and initial clinical presentation

On 12 September 2024, accidental leakage of waste liquids resulted in splatter exposure to the patient’s skin, oral cavity, and nasal passages. Subsequent symptom onset manifested as subjective discomfort within minutes, progressing to unconsciousness after a 20-min interval. Colleagues initiated immediate contact with emergency medical services, facilitating urgent transport to a local hospital. During the transfer, the patient experienced cardiac arrest and required cardiopulmonary resuscitation (CPR) for approximately 5–6 min before his heartbeat was restored. He arrived at the local hospital at 00:41 on 13 September 2024, for symptomatic treatment. Later that same day, at 3:36 a.m., he was transferred to our hospital due to a “disturbance of consciousness lasting 6 hours” for further diagnosis and treatment. Subsequent qualitative testing of the waste liquid provided by the factory confirmed that it contained nicotine and mescaline, which serves as a raw material in the synthesis of synthetic nicotine (Trotsko et al., 2024).

### 2.3 Key clinical findings on admission

Upon admission, the following physical examination results were recorded: temperature 36.4 °C; heart rate 112 beats per minute; respiratory rate 15 breaths per minute; and blood pressure 143/91 mmHg. The patient was in a comatose state and required mechanical ventilation. No jaundice was observed in the skin or mucous membranes, and the superficial lymph nodes were not enlarged. The left pupil measured approximately 3.5 mm in diameter, while the right pupil was about 4 mm, with both pupils showing no light reflex. No cyanosis of the lips, ulceration of the oral mucosa, or pharyngeal congestion was noted. The neck was soft and non-rigid. Bilateral thoracic movement was symmetrical, and breath sounds from both lungs were slightly coarse, with no dry or wet rales detected. The heart rate was regular at 112 beats per minute, with no pathological murmurs present in the valvular regions. The abdomen was flat, with no bowel sounds or peristaltic waves observed, and the tenderness examination was uncooperative. There were no spinal or extremity deformities. Muscle strength testing of the extremities was also uncooperative, but muscle tone appeared normal. The patient did not respond to painful stimuli, and no pathological reflexes were elicited. Arterial blood gas (ABG) analysis showed: pH 7.222, pCO_2_ 60.7 mmHg, pO_2_ 406.4 mmHg, lactate 5.7 mmol/L, blood glucose 14.3 mmol/L, and potassium (K^+^) at 3.03 mmol/L. Other abnormal laboratory results included: white blood cell count (WBC) 27.54 × 10^9^/L, neutrophil percentage (NEU%) 94.10%, lymphocyte percentage (LYM%) 1.90%, prothrombin time (PT) 14.60 s, and inflammatory markers: IL-6 31.90 pg/mL, I1B 5.35 pg/mL, I2R 290.00 u/mL, I-8 189.00 pg/mL, I-10 44.90 pg/mL, TNF-α 9.62 pg/mL, and lactate dehydrogenase (LDH) at 400 IU/L. Liver and kidney functions were within normal limits. The electrocardiogram revealed a mild rightward deviation of the electrical axis. The admission diagnosis was acute nicotine poisoning with subsequent cardiac arrest following CPR.

### 2.4 Hospital course and treatment

**FIGURE 2:**
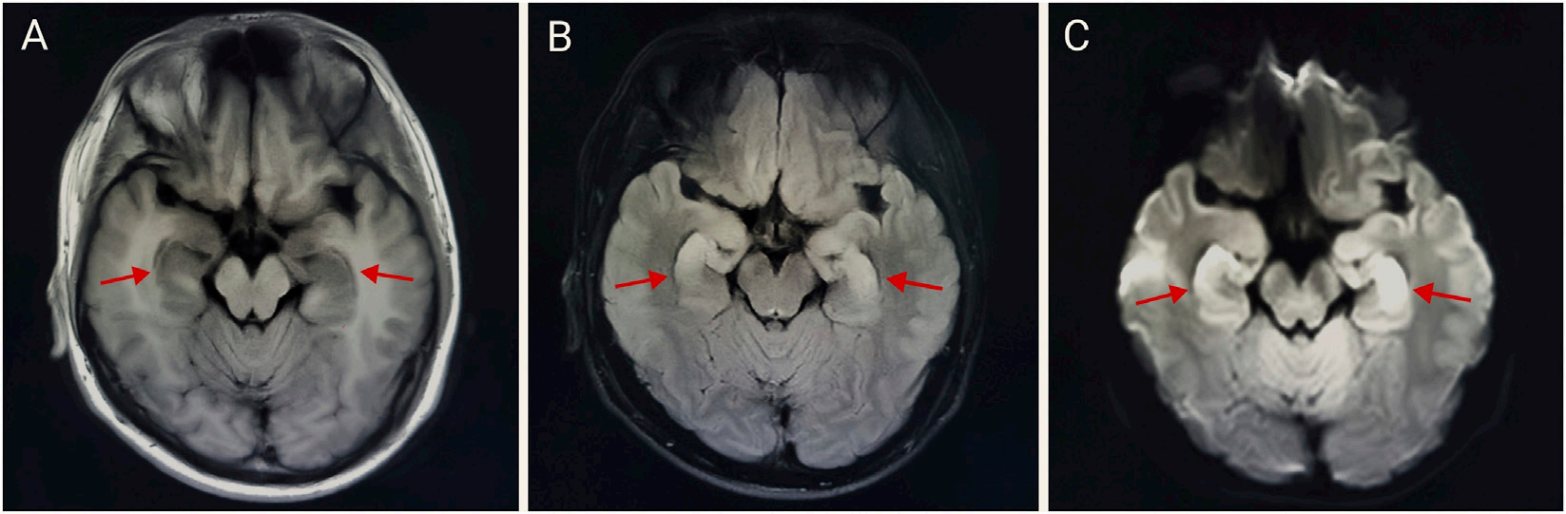
Bilateral hippocampal abnormal signal on 19 September 2024 **(A–C)**. Bilateral swelling in the hippocampus with hyperintensity on T2 and diffusion-weighted imaging (DWI). **(A)** T1-FLAIR, **(B)** T2-FLAIR, and **(C)** b = 1,000,DWI.

**FIGURE 3:**
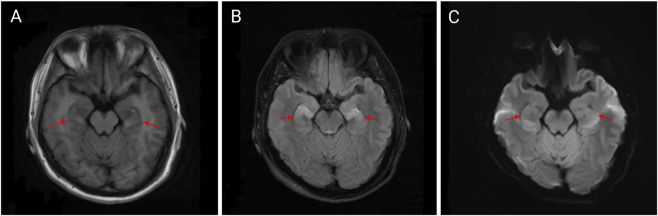
Bilateral hippocampal abnormal signal on 21 October 2024 **(A–C)**. Bilateral slight swelling in the hippocampus with hyperintensity on T2 and a slightly elevated DWI signal, similar to findings from 19 September 2024 **(A)** T1-FLAIR, **(B)** T2-FLAIR, and **(C)** b = 1000,DWI.

**FIGURE 4:**
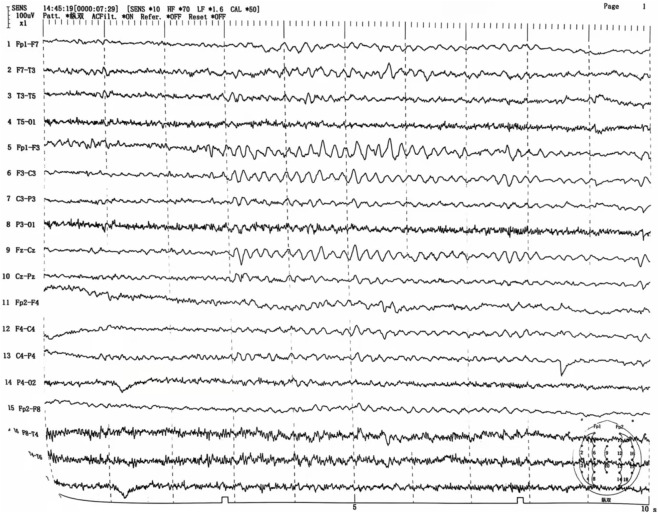
EEG on 21 October 2024 Moderately abnormal EEG with increased diffuse interstitial slow-wave activity, predominantly in the theta band, most pronounced in the left anterior head.

Upon admission, the patient received a comprehensive treatment regimen that included glucocorticoids, organ protection therapy, anti-infection measures, rehydration, and nutritional support. 3 h after admission, the patient experienced convulsions, which were alleviated with the administration of sodium valproate and sedatives. On hospitalization day 2, cranial, thoracic, and abdominal CT scans revealed no abnormal density foci in the brain parenchyma, no significant ventricular system enlargement, and no sulci widening. The midline structures were centered, although a few fibrous foci were noted in both lungs along with multiple dilated intestinal segments and effusions. On day 3 of hospitalization, the tracheal tube was removed, and the patient regained consciousness. Oxygen was provided *via* a nasal cannula. An MRI of the head and neck conducted on hospital day 7 showed bilateral swelling of the hippocampus with hyperintensity on T2 and diffusion-weighted imaging (DWI) (see Figure 2). On hospitalization day 22, an electroencephalogram (EEG) indicated moderate abnormalities, including a small amount of 7–9 Hz alpha rhythm in the occipital region and increased diffuse slow wave activity predominantly in the theta frequency band. Electromyography revealed decreased amplitude in recordings from the right common peroneal nerve’s phalangeal extensor; however, no abnormalities were found in other examined nerves or muscles. On hospitalization day 24, clinical symptoms were resolved significantly, leading to the patient’s discharge from the hospital. On 21 October 2024, routine blood tests and assessments of liver and kidney function were performed with no abnormalities detected. An MRI of the head and neck showed slight swelling of both hippocampi with hyperintensity on T2 and a slightly elevated DWI signal, similar to findings from 19 September 2024 (see Figure 3), while the EEG showed moderate abnormalities with increased diffuse slow wave activity, particularly pronounced in the theta band in the left anterior region (see Figure 4). The patient is currently under active follow-up, and findings warranting clinical significance will be compiled and reported in future publications.

## 3 Discussion

Nicotine, a toxic alkaloid present in tobacco, can be absorbed through multiple routes, including ingestion, inhalation, and dermal contact, resulting in a variety of adverse effects (Riordan et al., 2002). The lethal dose of nicotine in adults is a subject of debate; although the commonly accepted threshold is 60 mg, some studies suggest that the true lethal dose may exceed this amount (Solarino et al., 2010; Mayer, 2014). Previous case reports have shown that nicotine poisoning primarily occurs when children accidently ingest e-cigarette liquids or when adults consume these liquids for suicidal purposes (Becam et al., 2023). Occupational nicotine poisoning is relatively rare. The clinical manifestations of nicotine poisoning are mediated by nicotinic acetylcholine receptors (nAChRs), which are predominantly located in the central nervous system, autonomic nervous system, and various target organs (Dorotheo et al., 2024). Acute nicotine intoxication can mimic symptoms associated with bipolar disorder. In the early stages of intoxication or with minimal exposure, nAChRs become overstimulated, leading to cholinergic receptor overactivation. The most common symptom of this overactivation is tachycardia, which may be accompanied by other manifestations such as hypertension, shortness of breath, nausea, vomiting, excessive salivation, dizziness, confusion, and tremors (Maessen et al., 2020). Moreover, individuals exposed to high doses of nicotine may experience severe suppression of organ functions. This can manifest as drowsiness, convulsions, hypotension, coma, respiratory muscle paralysis, and even cardiac arrest following the initial stimulation phase (Henstra et al., 2022).

In this case, the patient was exposed to a significant amount of nicotine-containing waste liquid that sprayed onto his body, mouth, and nose during the production process. This exposure led to a rapid onset of coma and cardiac arrest. After receiving symptomatic and supportive treatment, the patient was discharged from the hospital 24 days later. To date, this is the first reported case of abnormal signal changes in the bilateral hippocampal region resulting from nicotine intoxication. MRI findings revealed bilateral hippocampal swelling with hyperintensity on T2 and diffusion-weighted imaging (DWI). These changes may be associated with ischemic-hypoxic brain damage following cardiac arrest, as the hippocampus is particularly vulnerable to ischemia and hypoxia (Greer et al., 2013). Alternatively, the observed alterations could be a result of neuronal cell death in the hippocampus induced by high doses of nicotine (Abrous et al., 2002).

This case demonstrates the potentially life-threatening consequences, including coma and cardiac arrest, that can arise within an extremely short time following a high-level occupational nicotine exposure. The distinctive neuroimaging findings—bilateral hippocampal swelling with hyperintensity on T2-weighted and diffusion-weighted imaging (DWI) —provide novel radiological evidence implicating either severe nicotine intoxication itself or post-cardiopulmonary resuscitation (CPR) ischemic-hypoxic brain injury affecting the hippocampus. These findings warrant further investigation into their underlying mechanisms and prognostic significance. The diagnostic process in this case underscores the critical importance of obtaining a detailed occupational and environmental exposure history during emergency evaluation, which is key to identifying the cause and initiating therapy. Therapeutically, management was complicated by post-resuscitation syndrome and the potential for severe neurological damage. The successful outcome in this case demonstrates that sustained intensive life support, timely targeted therapeutic interventions, coupled with long-term neurorehabilitation, are essential for the survival and recovery of patients suffering from such poisoning. As regulatory policies for e-cigarettes become increasingly stringent, synthetic nicotine has emerged as a new focus for the development of these products (Jordt, 2023). This near-fatal occupational poisoning incident serves as a stark warning for occupational health and safety. Companies involved should: implement stringent safety oversight measures in the production environment; enforce the mandatory use of enhanced personal protective equipment; equip workplaces with comprehensive emergency facilities (e.g., Automated External Defibrillators - AEDs); provide employees with targeted first-aid training to ensure effective immediate response following exposure; and conduct regular health checkups for employees. Additionally, in light of the rapid growth in e-cigarette use, relevant authorities should also strengthen regulation over the sales channels and market for e-cigarette liquids, standardizing safety protocols (Kim and Baum, 2015).

To our knowledge, this is the first reported case of bilateral hippocampal swelling with hyperintensity caused by acute nicotine poisoning. The unusual radiologic changes observed in this case were likely related to the massive nicotine exposure. Given the lack of established imaging diagnostic criteria for severe nicotine poisoning, these distinctive MRI features hold potential value for diagnosis and prognosis in life-threatening nicotine-induced cases.

## Data Availability

The original contributions presented in the study are included in the article/supplementary material, further inquiries can be directed to the corresponding authors.
